# Characterization of Cerebral Embolic Capture Using the SENTINEL Device During Transcatheter Aortic Valve Implantation in Low to Intermediate-Risk Patients: The SENTINEL-LIR Study

**DOI:** 10.1161/CIRCINTERVENTIONS.121.011358

**Published:** 2022-03-11

**Authors:** Rika Kawakami, Hemal Gada, Michael J. Rinaldi, Tamim M. Nazif, Martin B. Leon, Samir Kapadia, Amar Krishnaswamy, Atsushi Sakamoto, Yu Sato, Masayuki Mori, Kenji Kawai, Anne Cornelissen, Ji-Eun Park, Saikat Kumar B. Ghosh, Biniyam G. Abebe, Maria Romero, Renu Virmani, Aloke V. Finn

**Affiliations:** 1 CVPath Institute, Gaithersburg, MD (R.K., A.S., Y.S., M.M., K.K., A.C., S.K.B.G., B.G.A., M.R., R.V., A.V.F.).; 2 University of Pittsburgh Medical Center Pinnacle, Harrisburg, PA (H.G.).; 3 Sanger Heart and Vascular Institute, Atrium Health, Charlotte, NC (M.J.R.).; 4 Columbia University Irving Medical Center, New York City, NY (T.M.N., M.B.L.).; 5 Cleveland Clinic Foundation, OH (S.K., A.K.).; 6 Division of Cardiovascular Medicine, Department of Medicine, University of Maryland Medical Center (J.-E.P., A.V.F.).

**Keywords:** aortic valve, dilatation, incidence, ischemic stroke, transient ischemic attack

Indications for transcatheter aortic valve replacement (TAVR) have expanded to aortic stenosis patients with low- and intermediate-risk of surgery.^1^ Post-TAVR stroke causes acute and long-term morbidity and mortality. The stroke rate 30 days after TAVR was reported as 3.4% in low-risk patients.^2^ Prior studies including intermediate- and high-surgical risk cases suggested the use of the SENTINEL Cerebral Protection System (SENTINEL-CPS) during TAVR may reduce the incidence of ischemic stroke and in-hospital mortality.^3,4^ However, the effectiveness of the SENTINEL-CPS during TAVR in lower-risk patients has not been studied yet. The SENTINEL-LIR study (Cerebral Protection of Acute Embolic Burden During Transcatheter Aortic Valve Implantation in Low to Intermediate Risk Patients) aimed to quantify the frequency of embolic debris captured by the SENTINEL-CPS in lower-risk TAVR cases.

We will make the data available upon reasonable request. The SENTINEL-LIR registry was a multicenter, prospective clinical study (URL: https://www.clinicaltrials.gov; Unique identifier: NCT04131127). All patients provided written informed consent. The study protocol was approved by the institutional ethics review committee at each site. Patients with severe, symptomatic aortic stenosis, undergoing transfemoral TAVR with planned use of the SENTINEL-CPS, and a Society of Thoracic Surgeons Predicted Risk of Mortality score of <4% were included. Exclusion criteria were (1) a history of stroke or transient ischemic attack within 6 months of TAVR, (2) carotid artery intervention within 6 weeks of TAVR, (3) prior aortic valve replacement, or (4) concomitant surgical procedure. The clinical team decided the types of transcatheter heart valves implanted (CoreValve Evolut Pro, Medtronic; or Sapien 3, Edwards).The incidence of debris, size, and tissue types captured by the SENTINEL-CPS were histologically analyzed. The details of sample processing and analysis methods have been described previously.^4,5^

A total of 50 patients from 4 facilities in the US were enrolled (University of Pittsburgh Medical Center Pinnacle [n=26], Sanger Heart and Vascular Institute [n=14], Columbia University Irving Medical Center [n=7], and Cleveland Clinic [n=3]) between March and August 2020. One case was excluded due to valve-in-valve TAVR. Participants had a mean age of 76-years and 45% were women. The median Society of Thoracic Surgeons Predicted Risk of Mortality score was 1.7%, with 86% and 14% of participants classified by the heart team as low- and intermediate-risk, respectively.

Fifty-five percent of patients received the CoreValve, while 45% had theSapien 3. Predilatation and postdilatation were performed in 43% and 18% of cases, respectively. The frequency of predilatation and postdilatation were not different between the 2 transcatheter heart valves (predilatation: CoreValve 37% versus Sapien 50%, *P*=0.36; postdilatation: 15% versus 23%, *P*=0.48). Both proximal and distal filters of the SENTINEL-CPS were successfully placed in all patients. The SENTINEL-CPS was delivered and retrieved via radial access in 96%, brachial access in 2%, and ulnar artery approach in 2%. No vascular complications caused by upper extremity access were observed. The rates of death or stroke (as defined by the Valve Academic Research Consortium 2) within 30 days post-TAVR were 2% (1/49, due to myocardial infarction) and 4% (2/49), respectively. This study did not include routine brain imaging evaluation.

In histology, debris was captured in all 49 cases (100%). Debris was composed of arterial wall (98% of cases), acute thrombus (96%), valve tissue (71%), calcification (55%), and foreign materials (43%). Myocardial tissue (20%), organizing thrombus (4%), and necrotic core (4%) were found less frequently (Figure [A]).

**Figure.**
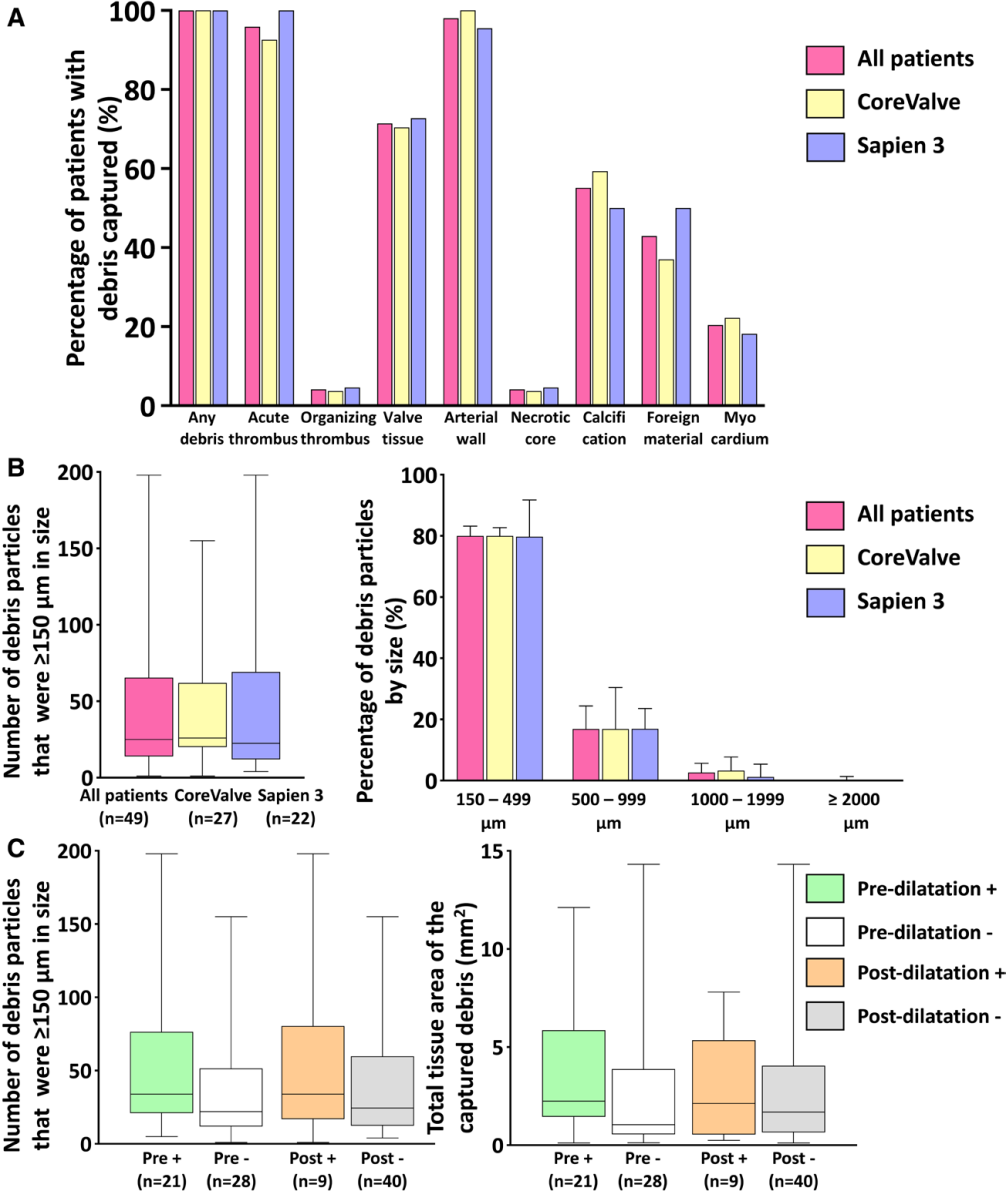
**Histological characteristics of debris captured by the SENTINEL cerebral protection system. A**, Debris capture rate for each tissue type. Debris was captured in all 49 patients (100%). The captured debris was composed of arterial wall fragments (98% of patients), acute thrombus (96%), valve tissue (71%), calcification (55%), and foreign materials (43%). Myocardium (20%), organizing thrombus (4%), and necrotic core (4%) were found less frequently. This tendency was identical in patients treated with CoreValve and Sapien 3 valves (arterial wall fragments: CoreValve 100%, Sapien 96%, *P*=0.26; acute thrombus: CoreValve 93%, Sapien 100%, *P*=0.19; valve tissue: CoreValve 70%, Sapien 73%, *P*=0.86; calcification: CoreValve 59%, Sapien 50%, *P*=0.52; foreign materials: CoreValve 37%, Sapien 50%, *P*=0.36; myocardium: CoreValve 22%, Sapien 18%, *P*=0.73; organizing thrombus: CoreValve 4% vs Sapien 5%, *P*=0.88; necrotic core; CoreValve 4%, Sapien 5%, *P*=0.88). **B**, Number of captured debris particles and percentages of captured debris by size. Median number of particles ≥150 μm in size was 25 (first–third quartiles, 14–67). No statistically significant differences in particle numbers and sizes were observed in patients implanted with CoreValve and Sapien 3 valves (all particle counts: CoreValve (median [first–third quartiles], 261 [152–407], Sapien 215 [150–620]; particles ≥150 μm in size: CoreValve 26 [20–62], Sapien 23 [12–69], *P*=0.49; total debris tissue area: CoreValve 2.25 [0.86–4.07], Sapien 1.28 [0.42–4.93], *P*=0.17). The majority of debris particles ranged in size from 150 to 499 μm (78% of particles were ≥150 μm in size); 17% of particles were 500–999 μm in size. Particles 1000–1999 μm and ≥2000 μm in size comprised 5% of the captured particles (100–1999 μm: 4%; ≥2000 μm: 1%). These observations were similar among patients implanted with CoreValve and Sapien 3 valves. Boxes with bars in the left graph indicate interquartile ranges corresponding to each median. Whiskers indicate the minima and maxima. Boxes with error bars in the right graph indicate medians and 75% quartiles. **C**, Effects of predilatation and postdilatation on captured debris. Performing predilatation or postdilatation did not impact the number of particles or the total tissue area of debris observed in the current study (number of particles ≥150 μm in size: predilatation, 34 [21–77], predilatation, 22 [12–52], *P*=0.06; postdilatation, 34 [17–81], postdilatation, 25 [13–60], *P*=0.40; total tissue area, predilatation, 2.25 [1.45–5.86], predilatation, 1.05 [0.56–3.88], *P*=0.05; postdilatation, 2.13 [0.54–5.35], postdilatation, 1.69 [0.65–4.06], *P*=0.74). Boxes with bars indicate interquartile ranges corresponding to each median. Whiskers indicate the minima and the maxima. Categorical variables were analyzed with the χ^2^ test or Fisher exact method. Non-normally distributed data were compared with the Wilcoxon rank-sum test. A *P* value <0.05 was considered significant. All statistical analysis was performed utilizing JMP software (version 15.0, SAS, Cary, NC).

Most captured debris had a size of <500 μm (78% were 150–500 μm). Nearly 5% of the captured particles were ≥1000 μm (Figure [B]), and these were detected in 67% of cases. Transcatheter heart valve type did not impact the debris tissue types, particle numbers, or sizes. Calcified particles were more common in patients treated with predilatation (71% versus 43%, *P*=0.046). However, predilatation or postdilatation did not alter particle numbers or sizes (Figure [C]).

Overall, the results of the present study are align with those of previous studies conducted in the high-surgical risk population, undergoing TAVR with the SENTINEL-CPS, regarding the capture rate of debris, observed tissue types, and size/distributions of debris.^4,5^

The current study is the first to explore the SENTINEL-CPS in low- or intermediate-risk patients. This study has several limitations. First, due to the small sample size registry, any associations between the incidence of post-TAVR stroke in this lower-risk cohort and the characteristics of debris captured by the SENTINEL-CPS could not be evaluated (all cases received the SENTINEL-CPS treatment). Second, the type of transcatheter heart valve was selected at the operators’ discretion, not assigned randomly. Thus, the superiority of transcatheter heart valves might not be evaluated precisely. These limitations will be best addressed in the future large-scale randomized studies.

In conclusion, the SENTINEL-LIR study demonstrates that embolic debris capture by the SENTINEL-CPS during TAVR in low- to intermediate-risk patients was similar to that in previous studies conducted among higher-risk patients. Larger size particles (≥1000 μm), which can cause significant vessel obstruction, were present in 67% of cases. These findings suggest lower-risk patients undergoing TAVR have potentially a similar embolic risk as high-risk patients as evidenced by embolic debris capture.

## Article Information

### Acknowledgments

We thank the participants and staff of this registry.

### Sources of Funding

This clinical study was funded by the Boston Scientific.

### Disclosures

CVPath Institute have received institutional research support from NIH-HL141425, Leducq Foundation Grant, 4C Medical, 4Tech, Abbott Vascular, Ablative Solutions, Absorption Systems, Advanced NanoTherapies, Aerwave Medical, Alivas, Amgen, Asahi Medical, Aurios Medical, Avantec Vascular, BD, Biosensors, Biotronik, Biotyx Medical, Bolt Medical, Boston Scientific, Canon,Cardiac Implants, Cardiawave, CardioMech, Cardionomic, Celonova, Cerus, EndoVascular, Chansu Vascular Technologies, Childrens National, Concept Medical, Cook Medical, Cooper Health, Cormaze, CRL, Croivalve, CSI, Dexcom, Edwards Lifesciences, Elucid Bioimaging, eLum Technologies, Emboline, Endotronix, Envision, Filterlex, Imperative Care, Innovalve, Innovative, Cardiovascular Solutions, Intact Vascular,Interface Biolgics, Intershunt Technologies, Invatin, Lahav, Limflow, L&J Bio, Lutonix, Lyra Therapeutics, Mayo Clinic, Maywell, MDS, MedAlliance, Medanex, Medtronic, Mercator, Microport, Microvention, Neovasc, Nephronyx, Nova Vascular, Nyra Medical, Occultech, Olympus, Ohio Health, OrbusNeich, Ossio, Phenox, Pi-Cardia, Polares Medical, Polyvascular, Profusa, ProKidney, LLC, Protembis, Pulse Biosciences, Qool Therapeutics, Recombinetics, Recor Medical, Regencor, Renata Medical, Restore Medical, Ripple Therapeutics, Rush University, Sanofi, Shockwave, SMT, SoundPipe, Spartan Micro, Spectrawave, Surmodics, Terumo Corporation, The Jacobs Institute, Transmural Systems, Transverse Medical, TruLeaf, University of California, San Francisco (UCSF), University of Pittsburgh Medical Center (UPMC), Vascudyne, Vesper, Vetex Medical, Whiteswell, WL Gore, and Xeltis. Dr Kawakami has received research grants from Japan Heart Foundation/Bayer Yakuhin Research Grant Abroad. Dr Gada has received honoraria from Medtronic, Abbott, Bard, Boston Scientific and is a consultant of Medtronic, Abbott, Bard, and Boston Scientific. Dr Rinaldi received research grant from Boston Scientific, honoraria from Abbott Vascular, Boston Scientific, Edwards and is a consultant of Abbott Vascular, Boston Scientific, and Edwards. Dr Nazif has received honoraria from Boston Scientific, Edwards LifeSciences, Medtronic, and Venus MedTech. Dr Leon has received the institutional grants for clinical research from Abbott, Boston Scientific, Edwards, and Medtronic. Dr Cornelissen receives research grants from University Hospital RWTH Aachen. Dr Virmani has received honoraria from Abbott Vascular, Biosensors, Boston Scientific, Celonova, Cook Medical, Cordis, CSI, Lutonix Bard, Medtronic, OrbusNeich Medical, CeloNova, SINO Medical Technology, ReCor Medical, Terumo Corporation, W. L. Gore, Spectranetics and is a consultant Abbott Vascular; Boston Scientific, Celonova, Cook Medical, Cordis, CSI, Edwards Lifescience, Lutonix Bard, Medtronic, OrbusNeich Medical, ReCor Medical, Sinomededical Technology, Spectranetics. Dr Finn has received honoraria from Abbott Vascular, Biosensors, Boston Scientific, Celonova, Cook Medical, CSI, Lutonix Bard, Sinomed, Terumo Corporation and is a consultant to Amgen, Abbott Vascular, Boston Scientific, Celonova, Cook Medical, Lutonix Bard, Sinomed, Surmodics, Terumo Corporation, W. L. Gore, and Xeltis. The other authors report no conflicts.
